# Effect of LLLT on endothelial cells culture

**DOI:** 10.1007/s10103-014-1650-0

**Published:** 2014-09-18

**Authors:** Krzysztof Góralczyk, Justyna Szymańska, Małgorzata Łukowicz, Ewelina Drela, Roman Kotzbach, Mariusz Dubiel, Małgorzata Michalska, Barbara Góralczyk, Andrzej Zając, Danuta Rość

**Affiliations:** 1 Department of Pathophysiology, Collegium Medicum in Bydgoszcz, The Nicolaus Copernicus University in Toruń (NCU), Ul. M. Skłodowskiej-Curie 9, 85-094 Bydgoszcz, Poland; 2 Department of Lasertherapy and Physiotherapy, Collegium Medicum in Bydgoszcz, The Nicolaus Copernicus University in Toruń (NCU), Ul. M. Skłodowskiej-Curie 9, 85-094 Bydgoszcz, Poland; 3 Department of Rehabilitation, Jozef Pilsudski University of Physical Education, Warsaw, Poland; 4 Department of Clinical Nursing, Collegium Medicum Bydgoszcz, The Nicolaus Copernicus University in Toruń (NCU), Ul. M. Skłodowskiej-Curie 9, 85-094 Bydgoszcz, Poland; 5 Department of Optoelectronics and Optical Radiation, The Technical University in Białystok, Białystok, Poland

**Keywords:** Low-level laser therapy, LLLT, Endothelial cells, VEGF, sVEGFR-1, sVEGFR-2

## Abstract

Growth factors as vascular endothelial growth factor (VEGF), produced by the endothelial cells, take an essential part in pathological and physiological angiogenesis. The possibility of angiogenesis modulation by application of laser radiation may contribute to the improvement of its use in this process. Thus, the aim of the study was to investigate the influence of low-level laser therapy (LLLT) on the proliferation of endothelial cells, secretion of VEGF-A and presence of soluble VEGF receptors (sVEGFR-1 and sVEGFR-2) in the medium after in vitro culture. Isolated human umbilical vein endothelial cells (HUVECs) were irradiated using a diode laser at a wavelength of 635 nm and power density of 1,875 mW/cm^2^. Depending on radiation energy density, the experiment was conducted in four groups: I 0 J/cm^2^ (control group), II 2 J/cm^2^, III 4 J/cm^2^, and IV 8 J/cm^2^. The use of laser radiation wavelength of 635 nm, was associated with a statistically significant increase in proliferation of endothelial cells (*p* = 0.0041). Moreover, at 635-nm wavelength, all doses of radiation significantly reduced the concentration of sVEGFR-1 (*p* = 0.0197).

## Introduction

The laser radiation produces many different effects depending on light-beam parameters as well as the tissues that are subjected to irradiation. Despite documented therapeutic effect of low-level laser therapy (LLLT), there is still much controversy and scientific debate over its effectiveness [1–3]. The variety of procedures and the inconclusive results of the conducted studies mobilized to search for the parameters of laser radiation, which both in vitro and in vivo will result in acceleration of cell proliferation and the expected therapeutic efficacy.

Laser radiation may affect individual cells only via the structures that absorb the radiation. Visible light photoreception (635 nm) occurs at the level of mitochondria [2, 4]. Consequently, electron transport, adenosine triphosphate nitric oxide release, blood flow, reactive oxygen species increase, and diverse signaling pathways are activated. Reports about this subject seem to confirm that the photoinducing electron transfer reactions may initiate the synthesis and conformational changes of proteins and may have an influence on DNA and RNA synthesis increase [4].

Laser therapy has a universal application in the wound healing, especially nonhealing decubitals and burned wounds, and also in the treatment of ulcer-dependent microcirculation disorders such as diabetic foot. Despite the proven therapeutic effect, there are still controversies about the mechanisms and nature of the interaction of laser radiation on living tissue. The literature analysis about this problem (this topic) demonstrates that the conducted studies were focused on keratinocytes and fibroblasts involved in tissue healing and only in a little extent on endothelial cells [5].

The endothelium plays many important functions in the human body. Maintaining vascular homeostasis, it plays a fundamental role in many pathological processes including hypertension, diabetes, or inflammatory process. It should be underlined that growth factors produced by endothelial cells (ECs) take a main part in tissue healing and angiogenesis process [6]. Wound healing is a multistep process and consists of these stages: primary hemostasis with platelets and secondary, leading to the production of fibrin, the contribution of inflammatory cells that migrate to the wound and cause inflammation, differentiation, proliferation, and migration of mesenchymal cells to the wound, angiogenesis, and reepitelialization.

Vascular endothelial growth factor (VEGF) is one of the key regulators of angiogenesis. The family of this growth factor includes VEGF-A, VEGF-B, VEGF-C, VEGF-D, VEGF-E, and PIGF (Eng. placental growth). The main proangiogenic factor—VEGF-A—occurs in several isoforms counting 121, 165, 189, and 206 amino acids. VEGF induces its biological effects by binding with high-affinity receptors belonging to the family of tyrosine kinase receptors [7]. Two known receptors for VEGF identified as VEGFR-1 (also known as FLT1) and VEGFR-2 (also known as KDR) are located in the endothelial cells of blood vessels. VEGFR-1 is responsible for the formation of capillary structures, while VEGFR-2 is expressed on endothelial cells involved in angiogenesis and circulating endothelial progenitor cells derived from the bone marrow [8]. The presence of soluble forms of receptors sVEGFR-1 and sVEGFR-2 with high affinity for VEGF was noted [9]. Both of the receptors, especially sVEGFR-1, are able to inhibit VEGF-induced mitogenesis and may be a physiological negative regulator of VEGF [10]. They reduce the availability of VEGF and prevent overgrowth of endothelial cells which is also used in a cancer therapy [11].

VEGF binding one of the receptors—VEGFR-2—initiates endothelial cell proliferation, which shows limited potential growth in the absence of stimulation by this factor. VEGFR-2 stimulation on mesodermal stem cells results in the transformation of these cells into endothelial cells. However, VEGFR-1 receptor is responsible for the formation of capillary structures. VEGF transmits a signal through the VEGFR-2 receptor, whose expression is increased on endothelial cells involved in angiogenesis and circulating endothelial progenitor cells derived from the bone marrow. Inhibiting VEGF or VEGFR-2 leads to apoptosis of endothelial cells and decreasing diameter, density, and vascular permeability [12, 13].

It is believed that VEGF plays a key role in postnatal, physiological (e.g., wound healing), and pathological (e.g., cancer, rheumatoid arthritis, proliferative retinopathy) angiogenesis [14]. To date, only few studies are concerned with the influence of LLLT on endothelial cell proliferation and VEGF-A secretion [15–17]. Thus, the aim of this study was to assess the impact of low-level laser radiation (LLLT) at a wavelength of 635 nm and 1,875 mW/cm^2^ power on vascular endothelial cells, secretion of VEGF-A, and presence of sVEGFR-1 and sVEGFR-2 receptors.

The aim of this study was to assess the impact of LLLT at a wavelength of 635 nm and power density of 1,875 mW/cm^2^ on vascular endothelial cells proliferation in vitro and secretion of VEGF-A and its receptors concentration.

## Material and methods

Endothelial cells (human umbilical vein endothelial cell (HUVEC) line) were derived from human umbilical veins by the enzyme method using collagenase according to the method described by Jaffe et al. [18]. Cells were cultured in M199 media supplemented with 20 % fetal bovine serum (FBS), 100 U/ml penicillin (Gibco^TM^) and growth factors: 50 μg/ml endothelial cell growth supplement (ECGS, Biomedical Technologies Inc. USA) and heparin. ECGS was obtained from a bovine hypothalamus. The cells of each treatment group and the control group were cultured in the same culture medium, which was changed before each irradiation. The cells were incubated at 37 °C in a humidified atmosphere with 5 % CO_2_. After two to four passage and seeding of the cells in 6-well culture plates, the proper experiment was conducted. HUVECs were plated at a density of 7.5 × 10^4^/cm^2^. Cells come from three independent isolations.

A semiconductor-based (GaAlAs) laser (Roithner Lasertechnik GmbH, Austria) was used to generate visible laser beam with the wavelength of 635 nm. In this paper, the authors have used the optoelectronic set for controlled, reproducible exposure of electromagnetic radiation of biological structures in the spectral band of tissue transmission window 600–1,000 nm [19]. At the cell-layer level, the power density measured using a laser power meter (Gentec, Model SOLO2 R2, Canada) was 1,875 mW/cm^2^ for 635 nm. The power was constant in all experiments. The distance between the laser source and the surface of application was 10 cm, application was carried through an optical fiber; the irradiated area was 80 cm^2^. The experiment was conducted in four groups: I the control group (no radiation); II the energy dose, 2 J/cm^2^; III 4 J/cm^2^; and IV 8 J/cm^2^. The cells were cultured for 6 days, with two radiations on the day nos. 2 and 4 with 1-day break. Conditioned medium from each well of culture plates was centrifuged for 10 min at 2,000×*g* and frozen at −86 °C. After thawing concentration of VEGF-A, sVEGFR-1 and sVEGFR-2 in the supernatant were measured by ELISA test (R&D company) according to the manufacturer’s instructions. The remaining cells on the bottom of each well were harvested using trypsin and counted by Buerker hemocytometry. This method uses trypan blue dye according to the method described by Basso et al. [20]. The results of the concentration of the parameters in the supernatant of each well culture plates were analyzed per number of cells in the each well.

Statistical analysis was performed using Statistical 9.1 for Windows (Stat Soft Inc.). The one-way ANOVA with post hoc test was used for the assessment differences between the control group and other research groups. Statistical significance was defined as *p* < 0.05.

The approval of the Bioethics Commission of the NCU Collegium Medicum in Bydgoszcz was obtained, No KB/135/2009, date 25.03.2009.

## Results

**Fig. 1:**
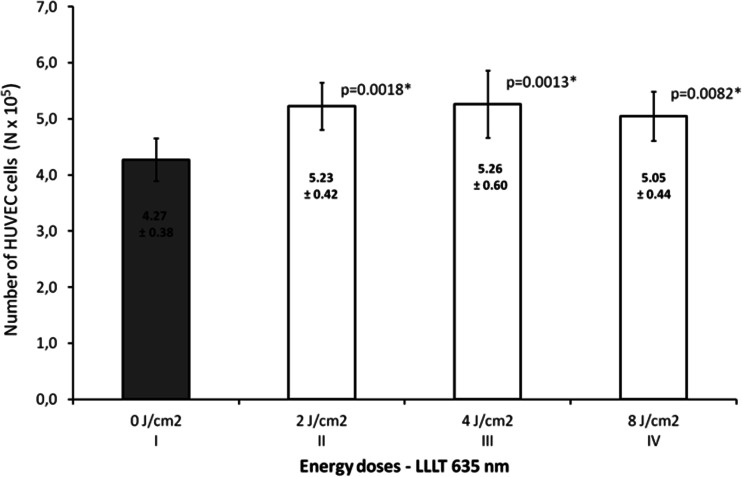
Number of HUVECs, depending on LLLT dose with the wavelength of 635 nm. Values are expressed as the mean ± SEM. *Asterisk above bars* indicate significant differences vs. control group (*p < 0.01)

**Fig. 2:**
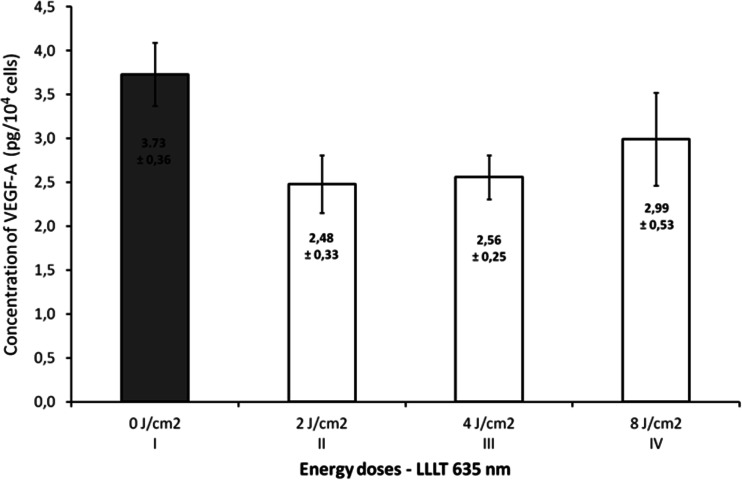
Concentration of VEGF-A in the supernatant, depending on LLLT dose with the wavelength of 635 nm (*p* = 0.08). Values are expressed as the mean ± SEM

**Fig. 3:**
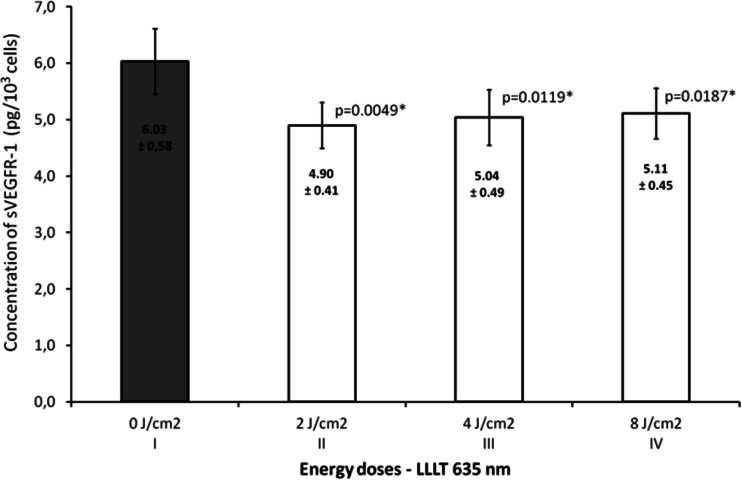
Concentration of sVEGFR-1 in the supernatant, depending on LLLT dose with the wavelength of 635 nm. Values are expressed as the mean ± SEM. *Asterisk above bars* indicate significant differences vs. control group (*p < 0.01)

**Fig. 4:**
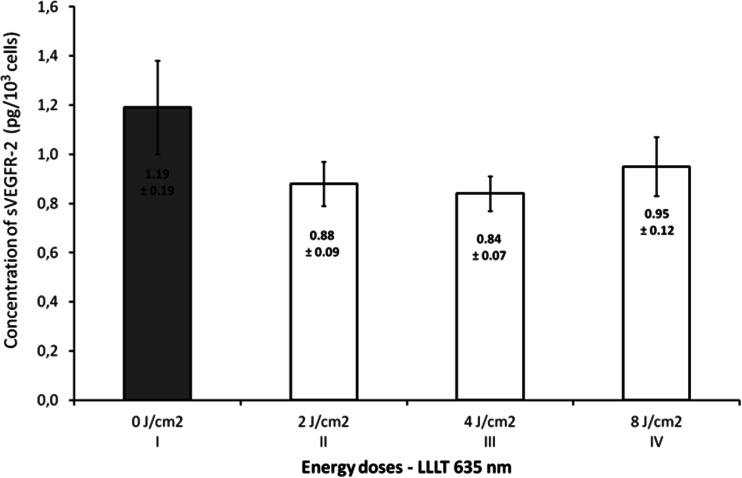
Concentration of sVEGFR-2 in the supernatant, depending on LLLT dose with the wavelength of 635 nm (*p* = 0.23). Values are expressed as the mean ± SEM

Figure 1 presents the effect of laser radiation with the wavelength of 635 nm (1,875 mW/cm^2^) on number of endothelial cells depending on rising energy dose of radiation. Energy doses of 2, 4, and 8 J/cm^2^ significantly increased number of cells (*p* = 0.0041, significant differences between groups: I vs. II, III, and IV). The highest value was observed at an energy dose of 2 and 4 J/cm^2^ and was about 23 % higher than in the control group. The concentrations of VEGF-A and its soluble receptors sVEGFR-1 and sVEGFR-2 are presented in Figs. 2, 3, and 4. The use of laser radiation of 635 nm (1,875 mW/cm^2^) was associated with a statistically significant lower concentration of sVEGFR-1. The results of analysis of variance were as follows: VEGF-A *p* = 0.0808, sVEGFR-1 *p* = 0.0197 (significant differences between groups I vs. II, III, and IV), and sVEGFR-2 *p* = 0.2340. Statistically significant *p* values for post hoc test were presented on the figures.

## Discussion

In the recent years, many studies have been concerned with angiogenesis process. VEGF is the major regulator of angiogenesis; thus in many studies, the attempt to explain the way of VEGF action through VEGFR-1 and VEGFR-2 was undertaken. The soluble forms of receptors for VEGF sVEGFR-1 and sVEGFR-2 are physiological inhibitors of EC proliferation. These receptors by binding with VEGF inhibit their action to stimulate cell proliferation and angiogenesis [11, 12].

In literature, there are a few references about influence of LLLT on VEGF secretion and angiogenesis [21, 22]. The authors, examining the processes of tissue healing with the use of LLLT, usually report the stimulation of fibroblast activity [23, 24]. Silva and colleagues [25] studied the expression of VEGF mRNA after irradiation LLLT wounds at rats. Using laser radiation with a wavelength at 780 nm, 70 mW power, and dose of 35 J/cm^2^ on the day of surgery and after 2 days (wavelength 660 nm, power 40 mW, dose of 5 J/cm^2^) showed its effect on the increased VEGF expression during the process of tissue healing.

Araujo and colleagues [23] conducted a detailed study of wound healing in mice after irradiation with He-Ne laser (632.8 nm) using the exposure parameters: 1 J/cm^2^ and exposure time 3 min. Autoradiographic and immunohistochemical analysis performed after 15 days of irradiation demonstrated reduction of inflammation, acceleration of reepitelialization, fibroblasts activation, and increased amounts of collagen fibers in the area of exposure. The results were compared with wounds that were not treated with LLLT.

**Fig. 5:**
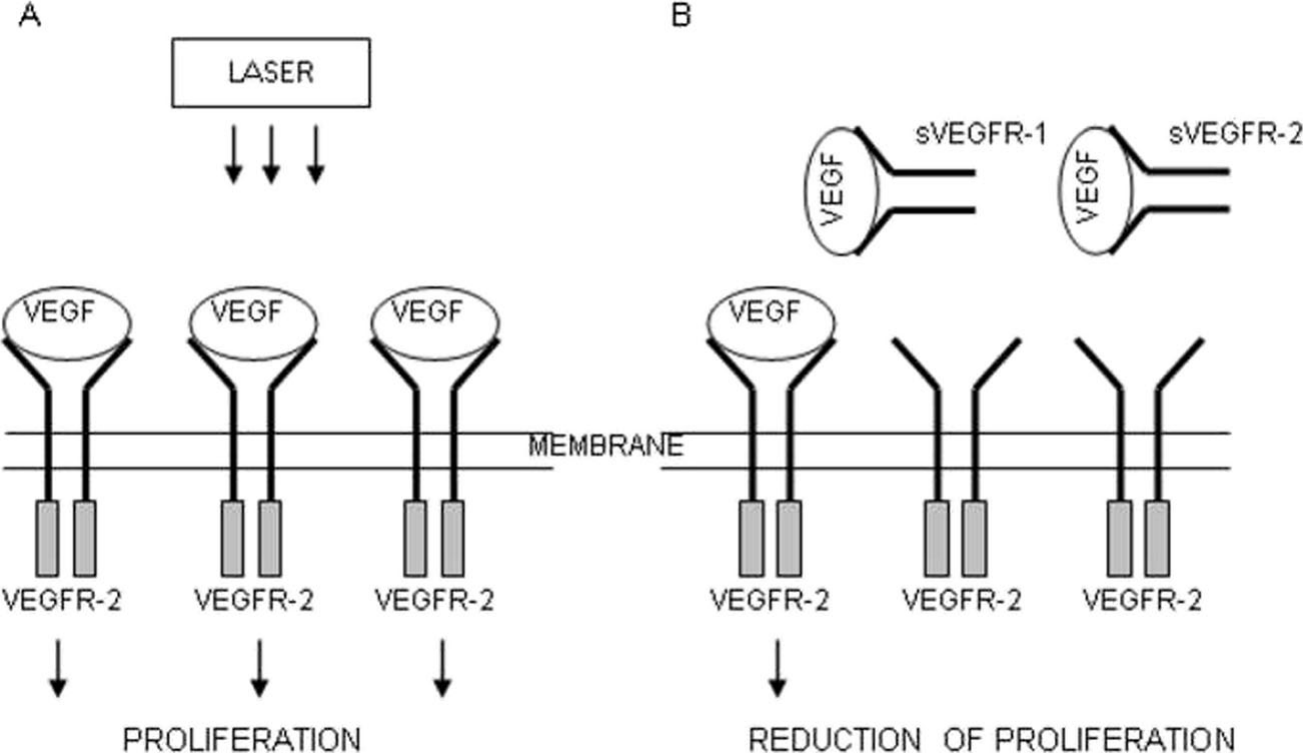
The hypothesis of the action of VEGF-A and soluble receptors sVEGFR-1 and sVEGFR-2 in the supernatant. **a** The connection of VEGF-A with VEGFR-2 receptor located on the cell membrane contributes to endothelial cell proliferation. **b** Competitive VEGF-A binding with soluble forms of receptors sVEGFR-1 and sVEGFR-2 present in the supernatant leads to reducing endothelial proliferation

In our study, varied effects of laser radiation on cells and secretion of angiogenic factors may be due to the different levels of photoreceptive interaction. The use of laser radiation in the visible light conditions resulted in the reduction of VEGF-A, sVEGFR-1, and sVEGFR-2 in the supernatant (Figs. 2, 3, and 4) with simultaneous activation of endothelial cell proliferation (Fig. 1). It is known that VEGF action is determined by VEGF binding with its membrane receptors. That causes receptors expenditure during the stimulation of endothelial cell division. In this way, the reduced amount of VEGF-A in the supernatant under the influence of laser radiation may be explained. The lowest VEGF-A concentration at doses 2 and 4 J/cm^2^ (*λ* = 635 nm) at simultaneously the highest level of cell proliferation suggests that in these conditions, the most molecules of VEGF-A are connected with receptors that significantly affected the level of EC proliferation. Lower concentrations of sVEGFR-1 and sVEGFR-2 in the supernatant as compared to the control group could contribute to the increase of cells proliferation by the influence of LLLT. Fewer VEGF-A molecules are blocked by soluble forms of receptors, which are regulators of VEGF [10, 11] (Fig. 5). This mechanism is used in cancer therapy, where the binding of VEGF and inhibiting VEGF native receptors suppress the new blood vessels creation, which reduces growth of the solid tumors and metastasis [11, 24]. In vitro studies conducted by Schindl et al. [25] confirmed the increase in HUVEC proliferation as a result of laser radiation at the wavelength range 670 nm and doses of 2, 4, 8 J/cm^2^.

This research should be continued, so that the influence of low-level laser radiation on endothelial cell proliferation and secretion of angiogenic factors is fully explained. The possibility of angiogenesis modulation by application laser radiation may contribute to the improvement of its use in the therapy of diseases whose base is the process of blood vessels formation (such as wound healing).

## Conclusions

1. Low-level laser therapy at 635 nm (1,875 mW/cm^2^) significantly increases the number of HUVECs.
2. Low-level laser therapy at 635 nm (1,875 mW/cm^2^) significantly decreases the concentration of soluble VEGFR- 1.

## References

[1] Leal-Junior EC, Vanin AA, Miranda EF, de Carvalho PD, Dal Corso S, Bjordal JM (2013) Effect of phototherapy (low-level laser therapy and light-emitting diode therapy) on exercise performance and markers of exercise recovery: a systematic review with meta-analysis. Laser Med Sci Nov 19 10.1007/s10103-013-1465-4 24249354

[2] Woodruff LD, Bounkeo JM, Brannon WM, Dawes KS, Barham CD, Waddell DL, Enwemeka CS (2004). The efficacy of laser therapy in wound repair: a meta-analysis of the literature. Photomed Laser Surg.

[3] Hawkins-Evans D, Abrahamse H (2008). Efficacy of three different laser wavelengths for in vitro wound healing. Photodermatol Photoimmunol Photomed.

[4] Karu T, Pyatibrat LV, Kalendo GS (2004). Photobiological modulation of cell attachment via cytochrome c oxidase. Photochem Photobiol Sci.

[5] Skopin MD, Molitor SC (2009). Effects of near-infrared laser exposure in a cellular model of wound healing. Photodermatol Photoimmunol Photomed.

[6] Aird WC (2007). Phenotypic heterogeneity of the endothelium: structure, function, and mechanisms. Circ Res.

[7] Crawford TN, Alfaro DV, Kerrison JB, Jablon EP (2009). Diabetic retinopathy and angiogenesis. Curr Diabetes Rev.

[8] Nowak K, Jachol N, Rafat N, Joas E, Beck GC, Hohenberger P (2013). Alterations of circulating bone marrow-derived VEGFR-2(+) progenitor cells in isolated limb perfusion with or without rhTNF-α. Ann Surg Oncol.

[9] Jain S, Ward MM, O’Loughlin J (2012). Incremental increase in VEGFR1 hematopoietic progenitor cells and VEGFR2 endothelial progenitor cells predicts relapse and lack of tumor response in breast cancer patients. Breast Cancer Res Treat.

[10] Ebos JM, Bocci G, Man S (2004). A naturally occurring soluble form of vascular endothelial growth factor receptor 2 detected in mouse and human plasma. Mol Cancer Res.

[11] Feldman AL, Libutti SK (2000). Progress in antiangiogenic gene therapy of cancer. Cancer.

[12] Roeckl W, Hecht D, Sztajer H, Waltenberger J, Yayon A, Weich HA (1998). Differential binding characteristics and cellular inhibition by soluble VEGF receptors 1 and 2. Exp Cell Res.

[13] Qazi Y, Maddula S, Ambati BK (2009). Mediators of ocular angiogenesis. J Genet.

[14] Messmer-Blust A, An X, Li J (2009). Hypoxia-regulated angiogenic inhibitors. Trends Cardiovasc Med.

[15] Szymanska J, Goralczyk K, Klawe JJ, Lukowicz M (2013). Phototherapy with low-level laser influences the proliferation of endothelial cells and vascular endothelial growth factor and transforming growth factor-beta secretion. J Physiol Pharmacol.

[16] Hou JF, Zhang H, Yuan X, Li J, Wei YJ, Hu SS (2008). In vitro effects of low-level laser irradiation for bone marrow mesenchymal stem cells: proliferation, growth factors secretion and myogenic differentiation. Lasers Surg Med.

[17] Kipshidze N, Nikolaychik V, Keelan MH (2001). Low-power helium: neon laser irradiation enhances production of vascular endothelial growth factor and promotes growth of endothelial cells in vitro. Lasers Surg Med.

[18] Jaffe EA, Nachman RL, Becker CG, Minick CR (1973). Culture of human endothelial cells derived from umbilical veins. Identification by morphologic and immunologic criteria. J Clin Investig.

[19] Gryko Ł, Gilewski M, Szymańska J, Zając A, Rość D (2012). The concept of the set to objectification of LLLT exposure. Proc. of SPIE: laser technology 2012. App Lasers.

[20] Basso FG, Oliveira CF, Kurachi C, Hebling J, Costa CA (2013). Biostimulatory effect of low-level laser therapy on keratinocytes in vitro. Laser Med Sci.

[21] Khanna A, Shankar LR, Keelan MH (1999). Augmentation of the expression of the proangiogenic genes in cardiomyocytes with low dose laser irradiation in vitro. Cardiovasc Radiat Med.

[22] Silva TC, Oliveira TM, Sakai VT (2010). In vivo effects on the expression of vascular endothelial growth factor-A165 messenger ribonucleic acid of an infrared diode laser associated or not with a visible red diode laser. Photomed Laser Surg.

[23] De Araujo CE, Ribeiro MS, Favaro R, Zezell DM, Zorn TMT (2007). Ultrastructural and autoradiographical analysis show a faster skin repair in He-Ne laser-treated wounds. J Photochem Photobiol B.

[24] Hornig CH, Barleon B, Ahmad S, Vuorela P, Ahmed A, Weich HA (2000). Release and complex formation of soluble VEGFR − 1 from endothelial cells and biological fluids. Lab Investig.

[25] Schindl A, Merwald H, Schindl L, Kaun C, Wojta J (2003). Direct stimulatory effect of low-intensity 670 nm laser irradiation on human endothelial cell proliferation. Br J Dermatol.

